# A guide to Direct Pathway Cloning (DiPaC) for natural product discovery

**DOI:** 10.1042/EBC20250016

**Published:** 2026-08-19

**Authors:** Lisa Göbner, Roberta R. de Castro, Tobias A. M. Gulder, Paul M. D'Agostino

**Affiliations:** 1 Department of Natural Product Biotechnology, Helmholtz Institute for Pharmaceutical Research Saarland (HIPS) and Helmholtz Centre for Infection Research (HZI), Campus E8.1, 66123, Saarbrücken, Germany; 2 Department of Pharmacy and PharmaScienceHub (PSH), Saarland University, Campus E8.1, 66123, Saarbrücken, Germany; 3 Chair of Technical Biochemistry, Technical University of Dresden, Bergstr. 66, 01069 Dresden, Germany

**Keywords:** biosynthetic gene cluster, cloning, molecular biology, natural products, synthetic biology

## Abstract

Natural products (NPs) are a major source of chemical diversity and bioactive molecules. Advances in genome sequencing and bioinformatic analysis have facilitated identification of biosynthetic gene clusters (BGCs) across diverse microorganisms and environments; however, experimental access to these pathways is often hindered by absence of genetic tools, limited availability of DNA, and complex genetic architectures. Direct Pathway Cloning (DiPaC) has emerged as an efficient and flexible synthetic biology approach to address these challenges. DiPaC combines long-amplicon PCR with *in vitro* DNA assembly, allowing simultaneous cloning and refactoring of biosynthetic pathways without the need for intermediate library construction, extensive *in vivo* recombination, or multiple antibiotic selection markers. The method provides broad flexibility in vector choice, host selection, and pathway architecture, facilitating promoter exchange, removal of inhibitory regulatory elements, and modular pathway reconstruction. This review highlights representative DiPaC applications spanning multiple NP classes and illustrates its utility in orphan BGC activation, probing biosynthetic logic, and combinatorial biosynthesis. In addition, we provide guidance on BGC selection, amplification of challenging sequences, and assembly strategies, addressing common technical obstacles. Future directions are discussed, positioning DiPaC as a cornerstone tool for modern NP research.

## Introduction

Natural products (NPs) are small organic molecules commonly produced by plants, bacteria, and fungi. Although not essential for survival, NPs can confer a significant fitness advantage through roles in competition, defence, and signalling [1]. In microorganisms, the genes responsible for encoding NPs are commonly co-localised on the genome into biosynthetic gene clusters (BGCs), a genomic feature that has become central to modern discovery efforts [2]. NP classes are commonly categorised by their associated biosynthetic logic, with the most prevalent including polyketide synthases (PKSs), non-ribosomal peptide synthetases (NRPSs), ribosomally synthesised and post-translationally modified peptides (RiPPs), terpenes, alkaloids, and combinations thereof [3]. BGCs typically encode different functional elements including core biosynthetic enzymes involved in core structure formation, precursor biosynthesis to supply specialised substrates, tailoring enzymes that modify the assembled scaffold, as well as regulatory, transport, or resistance proteins to regulate production, export, or confer self-resistance to the native producer, respectively [2].

The decreasing cost of whole-genome sequencing technologies has driven substantial growth in publicly available microbial genome data. In parallel, bioinformatic tools for detecting BGCs in assembled (meta-)genomes have established genome mining as a systematic approach for NP discovery [4–14]. Among these, AntiSMASH is currently the most widely used tool, capable of screening a sequenced genome and detecting well-characterised BGC types, leading to an ever increasing number of bioinformatically predicted cryptic BGCs. The scale of this approach is illustrated by the AntiSMASH database v5, which catalogues almost 479,420 BGC regions, of which the majority remain uncharacterised and represents a large reservoir of cryptic biosynthetic potential [15]. This has led to a paradigm shift where a major bottleneck in discovery is linking the functional characterisation of bioinformatically predicted BGCs to their encoded NPs. Unequivocally establishing the link between a BGC and its encoded NP requires either genetic manipulation in the native host or heterologous expression. Since many organisms lack established genetic tools, heterologous expression offers a flexible alternative and has driven the development of various BGC capture strategies coupled with expression systems [16–22]. These include *in vivo* and *in vitro* capture methods such as linear-linear and circular-linear homologous recombination as well as transformation-associated recombination cloning that exploit recombination machinery in *Escherichia coli* or *Saccharomyces cerevisiae*, respectively. Such methods are particularly well-studied to capture large linear genomic fragments of BGCs that exceed the practical limits of PCR amplification [21]. CRISPR–Cas-based methods, including CATCH, extend accessibility further by using *in vitro* Cas9 cleavage to excise the target cluster directly from intact chromosomal DNA embedded in agarose, capturing fragments of up to ∼100 kb in a single step [23] and Cas12a-based variants offer a related approach [24]. Similarly, advanced Cas9-mediated *in vivo* mobilisation and amplification of BGCs (ACTIMOT) allows CRISPR–Cas9-induced relocation and multiplication of BGCs onto multi-copy plasmids, resulting in increased gene dosage and elevated metabolite production [20,25]. While these methods can capture very large portions of genomic DNA (gDNA) the BGCs remain in their native configuration, with any desired refactoring carried out as a separate, post-capture step.

To add to the BGC capturing synthetic biology toolkit, we developed the Direct Pathway Cloning (DiPaC) strategy to streamline BGC cloning and refactoring. DiPaC combines long-amplicon PCR with *in vitro* DNA assembly to produce expression-ready constructs for further investigation by heterologous expression [26–29]. This review outlines the DiPaC strategy, representative applications, and common technical challenges to facilitate its broader application.

## Direct Pathway Cloning as an efficient strategy for BGC capture and refactoring

Selection of the BGC capturing strategy depends primarily on cluster size and the degree of refactoring required. DiPaC occupies a complementary position to other established methods: promoter exchange, removal of regulatory elements, and gene rearrangements are built into the primer design stage, integrating capture and refactoring into a single workflow, requiring neither intermediate library construction, yeast transformation, nor additional antibiotic selection markers. This makes DiPaC most practical for BGCs of small to medium size, typically up to ∼25 kb per amplification step, with larger clusters accessible through sequential assembly. Its principal limitations are dependence on PCR and high-quality gDNA, which becomes challenging for BGCs that are very large, highly repetitive, GC-rich, or have a complex transcriptional architecture (multiple putative operons or transcriptional terminators).

Central to DiPaC is the use of long-range PCR enabled by engineered polymerases with increased robustness and fidelity [26]. DNA assembly of PCR products with the vector backbone is mediated by either HiFi Gibson Assembly [30] or sequence- and ligation-independent cloning (SLIC) [28]. Because DiPaC directly amplifies BGCs from minimal quantities of DNA, it is applicable even when only small amounts of biological material are available. Vectors can be linearised either by preparative restriction digest or by PCR while the homology arms required for DNA assembly are appended using primers and incorporated during amplification. Because *in vitro* DNA assembly does not depend on host-specific recombination machinery, a wide range of vectors and expression hosts can be utilised, making DiPaC broadly flexible in its application.

**Figure 1:**
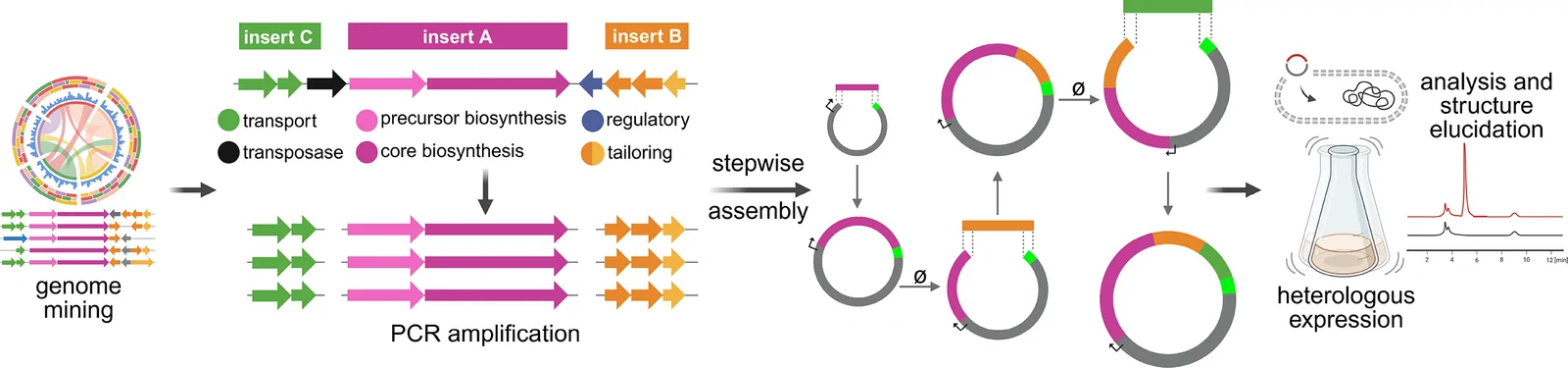
Overview of the DiPaC workflow BGCs of interest are subjected to a refactoring cloning strategy design that may include promoter replacement, removal of regulatory elements, or gene rearrangement. Selected regions are amplified by long-range PCR and assembled with an expression vector (grey, bright green: *gfp* transcriptional reporter) through *in vitro* DNA assembly. Large or complex BGCs can be assembled stepwise (sequential addition of fragments to a single plasmid) including linearisation steps (ø) in-between. Intermediate plasmid can also be used to probe biosynthesis *via* presence/absence of specific genes. The constructs are transformed into a heterologous host for functional studies.

The specificity of PCR-based amplification by primer design provides an opportunity to devise a cloning strategy to rationally design the reconstruction of a BGC of interest into the expression vector of choice [28]. For example, native promoters can be exchanged for well characterised promoters, transcriptional terminators and other detrimental regulatory genes or transposases can be removed, or genes encoded in different directions on the DNA can be assembled into a single polycistronic operon (Figure 1). Promoter exchange is important both when the source organism is phylogenetically distant from the heterologous host or when the native promoters are likely to be non-functional [31]. Importantly, multiple cloning steps do not require additional antibiotic cassettes for selection. Amplification of DNA fragments from gDNA up to 23.2 kb have been achieved [28] but PCR yields often decrease for larger or difficult templates. This potential issue can be bypassed by subdividing the BGC of interest into smaller fragments, which are subsequently re-assembled, either in a single step or over multiple DNA assembly rounds. For example, the 54.6 kb, high-GC erythromycin (*ery*) BGC from *Saccharopolyspora erythraea* NRRL 2338/DSM 40517 was divided into five fragments of 9.5–12.6 kb and assembled using a sequential DiPaC workflow [26]. A strategically introduced unique restriction at the 5′-end of each insert allowed repeated vector linearisation and addition of further fragments, producing a full-length *ery* construct. Overall, DiPaC provides a flexible platform for simultaneous cloning and refactoring of BGCs. Its applicability spans NP discovery, metabolic pathway characterisation and biosynthetic elucidation, knockouts and mutagenesis, and production optimisation.

**Figure 2:**
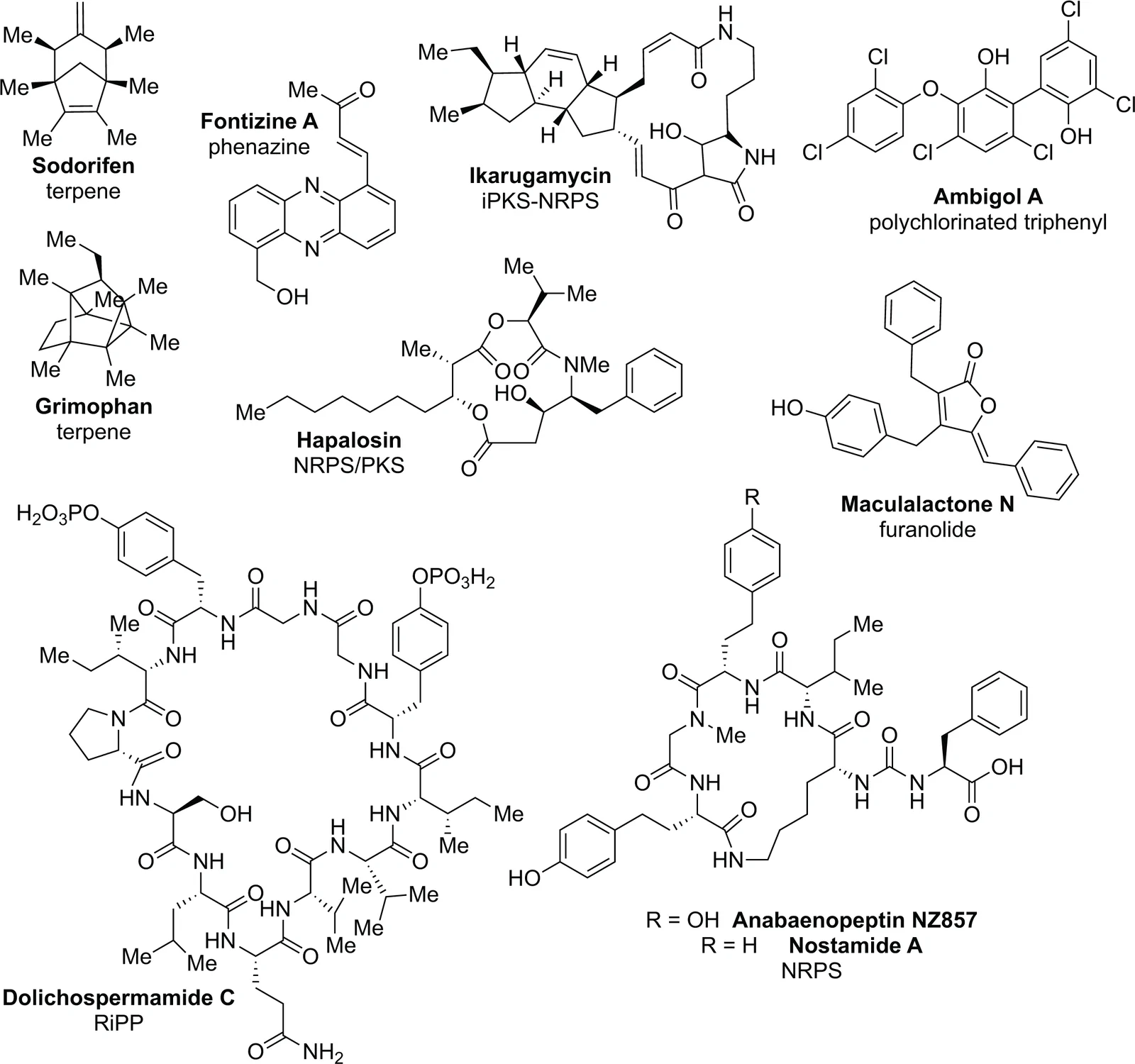
Selected examples of structural diversity DiPaC has been used to investigate a range of structural NP classes including NRPS, NRPS/PKS, *i*PKS, RiPP, polychlorinated triphenols, terpenes, phenazines and furanolides.

Application of the DiPaC strategy has led to the successful activation of BGCs of small to medium size sourced from different bacterial phyla and NP classes and has facilitated the connection of orphan or cryptic BGCs to their products (Table 1 and Figure 2). In *Serratia fonticola* DSM 4576, a silent phenazine BGC was identified bioinformatically and amplified as a single ∼10 kb fragment containing resistance, core, and tailoring genes [26]. Heterologous expression in *E. coli* BAP1 yielded two metabolites including the known 1,6-phenazinedimethanol and the new compound fontizine A, thereby linking this previously cryptic BGC to its products [26]. A similar strategy intercepted a small (∼4.6 kb) BGC from *Serratia plymuthica* WS3236, whose expression in *E. coli* BAP1 under the tetracycline-inducible *tetO* promoter confirmed its role in producing the highly unusual C_16_ volatile terpene sodorifen [32]. DiPaC also facilitated the discovery of structurally related sodorifen-type terpenoids from several *Pseudomonas* and enabled discovery and biosynthetic reconstitution of pseudorifen, aristotelene and the highly unusual C_17_ grimophan [33,34]. Likewise, DiPaC enabled the cloning and expression of the ∼13 kb ambigol (*ab*) BGC from the cyanobacterium *Symphyonema bifilamentata* 97.28 (formerly *Fischerella ambigua* 108b [35]), resulting in heterologous production of the polychlorinated triphenyl ambigol A and 2,4-dichloro-6-(2,4-dichlorophenoxy)-phenol in the host *Synechococcus elongatus* PCC 7492. This ultimately provided a platform for elucidating their unique biaryl-coupling chemistry [36], and also led to additional biosynthetic work and in-depth studies of the pharmaceutical potential of this compound class [37,38].

**Table 1:** Comprehensive overview of NPs accessed through DiPaC-mediated heterologous expression

Compound(s)	NP class	BGC size (kb)	Source organism(s)	Expression vector system(s)	Expression host(s)	Key cloning/ refactoring steps	Reference
Fontizine A 1,6-Phenazine- dimethanol	Phenazine	9.7	*Serratia fonticola* DSM 4576	pET28b-ptetO::*gfp*V2	*E. coli* BAP1	Native promoter replacement.	[26]
Erythromycin A	PKS	54.6	*Saccharopolyspora erythraea* NRRL 2338/DSM 40517	pLK01^▪^	*S. coelicolor* M1152 and M1154	Native promoter replacement.	[26]
Anabaenopeptin NZ857 Nostamide A	NRPS	29.2	*Nostoc punctiforme* PCC 73102	pET28b-ptetO::*gfp*V2 pETDuet-1	*E. coli* BAP1	Native promoter replacement. Precursor biosynthesis co-expressed.	[26]
Radiosumin D	NRPS	16.8	*Dolichospermum planctonicum* UHCC 0167	pET28b-ptetO::*gfp*V2	*E. coli* BAP1	Native promoter replacement. Two constructs generated: one with the complete BGC and second one with the addition of MATE efflux transporter *radI*.	[50]
Bacillamide D	NRPS	9.5	*Bacillus cereus* DSM 28590	pET28b-ptetO::*gfp*V2	*E. coli* BAP1	Native promoter replacement. Predicted *Rho*-independent terminator removed.	[29]
Bilothiazole A-F	PKS-NRPS	28.4	Uncultured *Bilophila* sp.^▴^	pET28b-ptetO::*gfp*V2	*E. coli* BAP1	Native promoter replacement. Commercial synthetic DNA with codon optimisation for expression in *E. coli*. Omission of two genes from the native BGC (*bilC* and *bilD*).	[39]
Hapalosin A	PKS-NRPS	25.7	*Fischerella* sp. PCC 9431	pET28b-ptetO::*gfp*V2	*E. coli* BAP1	Native promoter replacement. Predicted *Rho*-independent terminator removed. Intergenic region deletion.	[28]
Microginin family	PKS-NRPS	25	*Microcystis aeruginosa* LEGE 91341	pET28b-ptetO::*gfp*V2	*E. coli* BAP1	Native promoter replacement.	[51]
Ikarugamycin	*i*PKS-NRPS (PoTeM)	12.3	*Streptomyces* sp. Tü 6239	pSET152_ermE*, pUWL201PW, pWHM4* and pWHM1120	*S. albus* DSM 40313, *S. lividans* TK24, *S. coelicolor* M1154.	Native promoter replacement.	[44]
Ikarugamycin Capsimycin B, G Clifednamide A, C Butremycin.	*i*PKS-NRPS (PoTeM)	NA	*Streptomyces* sp. Tü 6239, *Streptomyces* sp. JV178^▴^, *S. pactum* SCSIO 02999^▴^	pSET152_ermE*	*S. albus* DSM 40313, B2P1 and Del14, *S. lividans* TK24, *S. coelicolor* M1154.	Native promoter replacement. Synthetic promoter library screening. Combinatorial expression of genes from different PoTeM BGCs. Establishment of a modular ‘plug-and-play’ cassette.	[43].
Dolichospermamide A-C	RiPP (Cyanobactin)	11.2	*Dolichospermum* sp. LEGE 00240	pET28b-ptetO::*gfp*V2	*E. coli* BL21(DE3)	Native promoter replacement. Generation of constructs with site-directed mutagenesis of the DolE2 core peptide for phosphorylation studies. Kinase domain from *dolG* removed.	[41]
Sphaerocyclamide family	RiPP (Cyanobactin)	10.3	*Sphaerospermopsis* sp. LEGE 00249	pET28b-ptetO::*gfp*V2	*E. coli* BL21(DE3)	Native promoter replacement. Engineering of the *sph* BGC by inserting the kinase domain from *dolG* (position 1294-2322 bp) into *sphG*.	[41]
							
Ambigol A 2,4-dichloro-6- (2,4-dichlorophenoxy)- phenol	Polychlorinated triphenyl	14.3	*S. bifilamentata* sp. nov. 97.28 (previously *F. ambigua* 108b)	pAM5051^▪^ and pCV0094^▪^	*S. elongatus* PCC 7942	The *ab* BGC was split into two modules for sequential chromosomal integration in *S. elongatus* PCC 7942.	[36]
Sodorifen	Terpene	4.6	*S. plymuthica* WS3236	pET28b-ptetO::*gfp*V2	*E. coli* BL21	Native promoter replacement.	[33]
Pseudorifen	Terpene	2.6	*Pseudomonas chlororaphis* DSM 6698	pET28b-ptetO::*gfp*V2	*E. coli*BAP1	Native promoter replacement. Six constructs generated: one full length *pcau* BGC and five variants with sequential gene removal to identify essential genes.	[33]
Aristotelene	Terpene	9.8	*Pseudomonas chlororaphis* DSM 50083	pET28b-ptetO::*gfp*V2	*E. coli* BAP1	Native promoter replacement. Three constructs generated: full length *pcch* BGC and two variants with sequential gene removal to identify essential genes.	[33]
Grimophan	Terpene	6.5	*Pseudomonas grimontii* DSM 17515	pET28b-ptetO::*gfp*V2	*E. coli* BAP1	Native promoter replacement. Multiple constructs generated with different gene arrangements to investigate biosynthetic route.	[34]
Precyanobacterins I-III (Cyanobacterin intermediates)^•^	Furanolide	15.7	*Tolypothrix* sp. PCC 9009	pET28b-ptetO::*gfp*V2	*E. coli* BAP1	Native promoter replacement. Predicted *Rho*-independent terminator and other intergenic regions removed; modular construct design for functional characterisation.	[27]
Maculalactone B Maculalactone N Deoxyenhygrolide A Other analogues^★^	Furanolide	15.5	*Nodularia* sp. NIES-3585	pET28b-ptetO::*gfp*V2	*E. coli* BAP1	Native promoter replacement. Two constructs generated: full length *mac* BGC and minimal *macBCEF* for functional characterisation and comparison.	[46]

▴ Metagenome-assembled genome BGC data of *Bilophila* sp. used as templates for commercial DNA synthesis.

▪ Vectors used for chromosomal integration of the BGC into the expression host.

• The final cyanobacterin structure could not be obtained under the expression conditions tested; only intermediates were detected.

★ An additional 22 compounds were identified and their structure predicted based on liquid chromatography–tandem mass spectrometry.

Refactoring during cloning is essential when regulatory elements prevent successful expression as shown in *B. cereus* DSM 28590, where DiPaC was used to clone a cryptic 9.5 kb NRPS predicted to encode a thiazoline-containing compound. Removal of a *Rho*-dependent terminator during DNA assembly allowed unhindered transcription, leading to production of the cytotoxic bacillamide D [29]. Even cryptic BGCs from metagenomes were accessed through DiPaC such as the bilothiazole BGC (∼28.4 kb) from an uncultured *Bilophila* sp. Commercially synthesised and codon-optimised DNA fragments were assembled using DiPaC, enabling the discovery of bilothiazoles A-F [39]. A refactoring approach was also vital to resolve the conflicting open reading frame (ORF) annotations between homologous hapalosin (*hap*) BGCs of *Fischerella* sp. PCC 9431 and *Hapalosiphon welwitschia* IC-52-3 [28,40]. The BGC harboured conflicting ORF annotations for *hapD* across different cyanobacterial genomes, with start codon positions differing by 180 bp. DiPaC enabled construction of two expression vectors carrying either the full-length or truncated *hapD*, with only the former yielding hapalosin and thus confirming the correct start codon [28].

Besides the capture and expression of cryptic BGCs, DiPaC has provided avenues for exploitation of NP biosynthetic logic (Table 1). The cyanobactin-like RiPPs from the cyanobacteria *Dolichospermum* sp. LEGE 00240 (*dol*, 11.2 kb) and *Aphanizomenon* sp. PH219 (*aph*, 10.4 kb) were captured and expressed in *E. coli* BL21, yielding the phosphorylated cyclic peptides dolichospermamide A-C and aphanizomenamide A-B, respectively [41]. The pathways encoded a peptidase/macrocyclase (*dolG*/*aphG*) harbouring an embedded rare kinase domain responsible for Tyr phosphorylation [41]. Transplantation of this domain into the canonical peptidase/macrocyclase *sphG* of the sphaerocyclamide BGC, resulted in phosphorylated derivatives.

Ikarugamycin belongs to the polycyclic tetramate macrolactam (PoTeM) family of NPs. The common tetramic acid precursor of all PoTeMs is assembled by the iterative PKS/NRPS (*i*PKS/NRPS) *ikaA*. Several oxidoreductases encoded in PoTeM BGCs perform various cyclisations on this substrate resulting in individual ring structures (e.g. IkaB and IkaC lead to the 5-6-5-cyclisation pattern of ikarugamycin), with further structural diversity installed by various tailoring enzymes [42]. A DiPaC-supported plug-and-play expression system containing *ikaABC* under the control of the constitutive ermE* promoter with screening of eight downstream promoters was used to optimise pathway expression across diverse *Streptomyces* hosts [43,44]. The downstream promoters were utilised for combinatorial incorporation of genes encoding PoTeM tailoring enzymes such as the hydroxylase PtmD and the monooxygenases IkaD and CftA, allowing systematic probing of post-assembly scaffold modifications and generation of structural derivatives. Mutagenesis within the adenylation domain of *ikaA* even enabled the production of the first analogue with an extended macrolactam structure [45]. (Table 1).

Cyanobacterin is a potent phytotoxin with a highly functionalized γ-butyrolactone core. DiPaC enabled the capture and refactoring of the 15.7 kb *cyb* cluster by removing predicted transcriptional terminators and other intergenic regions [27]. Amplification and DNA assembly of the expression vector led to multiple knockout plasmids to probe the core biosynthetic genes and thus uncovering the unprecedented enzymatic cyclisation chemistry in furanolide biosynthesis [27]. DiPaC has further expanded the known furanolide genetic diversity with the discovery of the silent maculalactone (*mac* 15.5 kb) BGC from the cyanobacterium *Nodularia* sp. NIES-3585 [46]. The furanolide biosynthetic logic was further exploited *in vivo* and *in vitro* through combinatorial constructs drawing on genes from the cyanobacterin, maculalactone, and scytonemin BGCs, yielding libraries of unnatural furanolides for bioactivity testing [27,46–49].

## A guide to the successful application of Direct Pathway Cloning

### Design of the BGC cloning strategy

**Figure 3:**
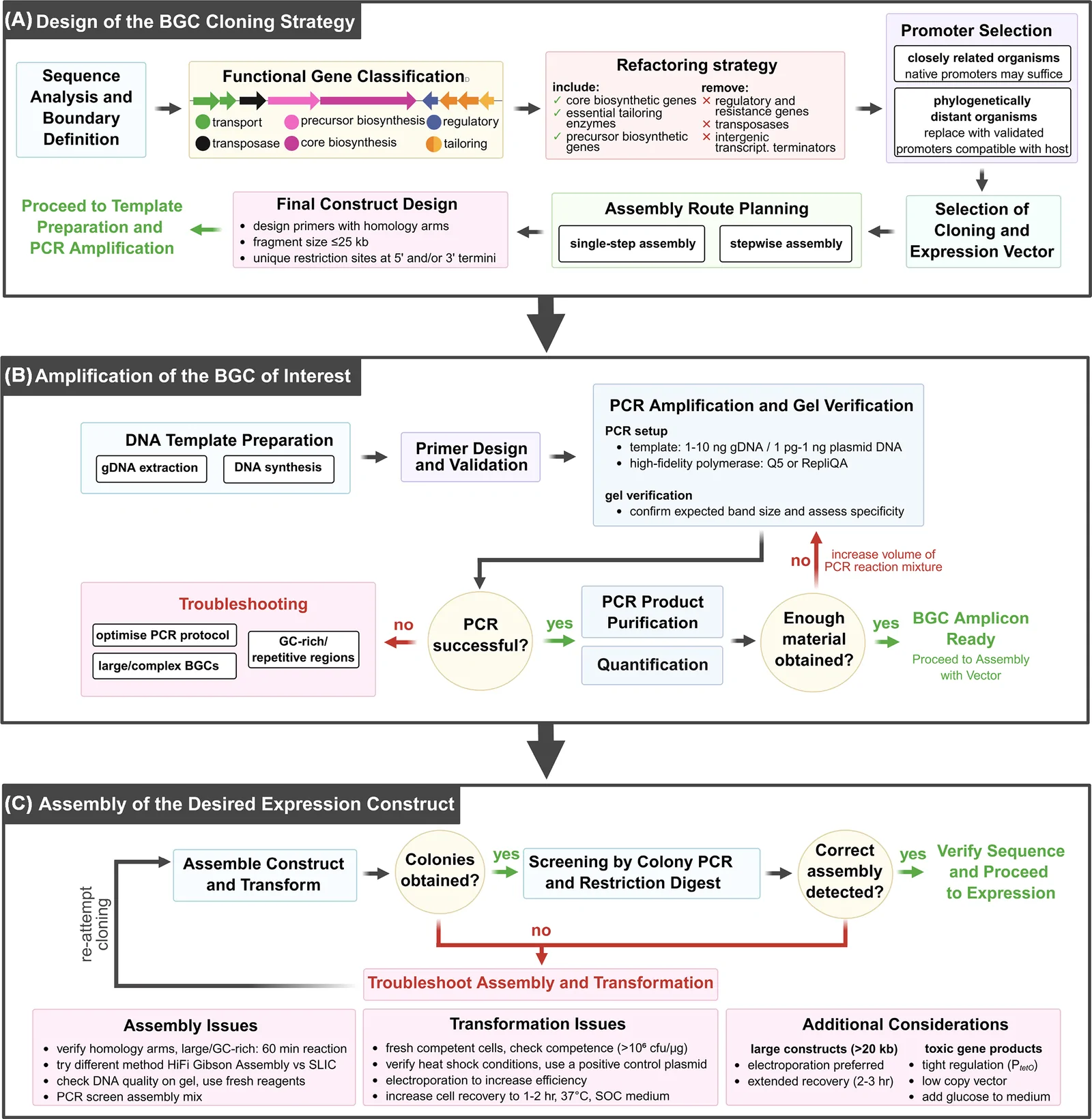
Flowchart of the DiPaC strategy and approaches to overcome common technical difficulties. Flowchart of the DiPaC strategy and approaches to overcome common technical difficulties. (**A**) Design of the BGC cloning strategy. (**B**) Amplification of the BGC of interest. (**C**) Assembly of the desired expression construct.

Key design decisions for the cloning strategy directly influence downstream experimental success (Figure 3). Important considerations include the BGC size and the complexity of the genetic organisation. Different organisms and BGC classes may possess varying degrees in operon complexity. In general, more complex BGCs, particularly those with multiple operons or transcriptional terminators, typically require additional cloning steps. Thus, bioinformatic analysis and theoretical design of the cloning strategy are the initial steps for capturing your BGC of interest.

Searching for alternative ORFs is also important when analysing BGCs, as highlighted by the incorrectly annotated *hapD* of *Fischerella* sp. PCC 9431 [28]. Software tools such as Geneious [52], SnapGene [53], Benchling (https://benchling.com) and related genome visualisation and analysis platforms can predict ORFs and help distinguish genuine coding sequences from extended reading frames. Genome visualisation tools can also be used to decipher the boundaries of a BGC of interest. Tools such as cblaster [54] and clinker [55] can be used to identify and align homologous BGCs. Comparative analysis of the aligned BGCs may help to discern the BGC boundaries based on sequence similarity and gene synteny. BGCs may sometimes possess GC-content patterns that differ from the surrounding genomic sequence, which can also be helpful to distinguishing BGC boundaries. Considering comparative genomics and GC profiles alongside ORF predictions can strengthen confidence in gene assignments and BGC borders allowing selection of genes for subsequent cloning.

Once the BGC of interest has been identified and the boundaries designated, the putative biosynthetic function of each gene (core biosynthesis, tailoring, etc.) are assigned using sequence homology tools such as NCBI BLAST (Basic Local Alignment Search Tool) [56] and domain annotation databases including NCBI Conserved Domain Database [57]. In general, core biosynthetic genes should be cloned first followed by precursor, tailoring or transporter genes whilst others that may be detrimental to biosynthesis or cloning, such as regulatory genes or transposases, are not included in the final expression vector. Another approach is to clone non-core biosynthetic genes on an additional plasmid harbouring a compatible origin of replication, as was performed for the biosynthetic precursor genes involved in homo amino acid biosynthesis of the anabaenopeptins [26]. In this way, intermediate plasmids may also probe biosynthesis *via* the presence/absence of specific tailoring enzymes. The next step is to remove other regulatory elements that may inhibit successful expression and thus, transcriptional terminators are predicted using the tools ARNold for *Rho*-independent terminators [58] or RhoTermPredict [59] for *Rho*-dependent terminators when using *E. coli* or *Bacillus* sp. as a heterologous host. Any putative transcriptional terminators identified in the intergenic regions of the BGC can then be removed using the targeted position of primers.

Overlapping CDSs are widespread across all domains of life and can enhance translational coupling, influence RNA structure, and support proper assembly or function of multi-component protein systems [60]. Because such overlaps can carry functional significance, their presence is an important genomic feature to be aware of in multi-step cloning strategies. Studies in which natural overlaps were removed show that even when individual protein levels remain stable, regulatory balance or complex integrity can be affected [60,61]. Thus, it is suggested to retain overlapping CDS when transferred to an expression plasmid whenever possible [60].

With the sections of the BGC defined, their theoretical reconstruction into the expression vector can be planned. The same commercial platforms mentioned above (Geneious [48], SnapGene [49], and Benchling) alongside dedicated tools such as the New England Biolabs NEBuilder^®^ (proposes primers based on your assembly and is highly recommended for those new to DNA assembly), in addition to j5 [62]and pydna [63], are generally sufficient for planning DiPaC constructs, as they allow the user to design primer positions, model homology arms, and simulate the assembled operon within a single environment.

### Amplification of the BGC of interest

The extraction of high-quality gDNA can strongly influence the success of all downstream amplification and assembly steps. Achieving efficient and complete cell lysis while preserving intact and pure gDNA for an organism is important for downstream amplification. The increasing reliance on metagenomic datasets for discovering BGCs further complicates gDNA preparation, since they often yield highly fragmented and impure gDNA. As a result, synthetic DNA is becoming an increasingly attractive alternative for accessing BGCs that are difficult to isolate or remain unavailable, even though synthesis costs, particularly for larger constructs, remain a significant consideration. However, purchasing smaller synthetic constructs which are later used as a template for DiPaC-mediated construction of the expression plasmid is a possible and cost-efficient solution.

Successful amplification relies on careful primer design [64]. Primers should incorporate stabilising terminal 3′ G/C nucleotides (GC clamp) and avoid repetitive or overly common sequence regions in addition to extended homopolymeric G/C tracts, if possible. For DiPaC, primers additionally incorporate homology arms to direct *in vitro* assembly, which should have similar length and Tm to the annealing region and are generally better placed on the smaller amplicon (insert or vector-backbone) to minimise effects on amplification efficiency. The Tm of primer incorporated homology arms should be suitable for annealing of terminal DNA strands at the desired temperature of the assembly reaction. For example, if the annealing temperature of the homology regions are lower than the DNA assembly reaction, than annealing of DNA fragments cannot occur and cloning is likely to fail. Primers should be evaluated for dimerisation, secondary structure, hairpin formation, Tm discrepancies or potential off-target amplification using available online tools such as NEB Tm Calculator [65], Sigma OligoEvaluator™ [66], IDT OligoAnalyzer™ [67] or Primer-BLAST [68]. If PCR fails, troubleshooting should begin with optimisation of PCR conditions, including assessment of template quality and concentration; high-quality gDNA at sufficient stock concentration is important to allow adequate dilution, as this reduces the carry-over of co-purified contaminants. gDNA requires higher input amounts than plasmid DNA, though excessive DNA template can inhibit PCR reactions, and we found concentrations of 1–10 ng per reaction are optimal. Adhering to polymerase-specific recommendations is essential; enzymes such as Q5 and RepliQA have performed reliably in high-fidelity applications in our hands. Thermocycler optimisation—including gradient PCR to empirically identify the optimal primer annealing temperature, especially for primers that include homology arms, increasing extension times up to 1 min/kb, and lengthening final extension time—can further improve yields. If these steps fail, primer redesign should be considered. As any PCR-based method is inherently susceptible to the introduction of mutations, whole-plasmid sequencing of final constructs is recommended.

In cases where the BGC exhibits complex organisation or dispersed genomic arrangement, overlapping extension PCR [69] can further facilitate the consolidation of multiple PCR products into a single contiguous DNA fragment, subsequently improving cloning efficiency. This approach is particularly valuable for BGCs that harbour multiple transcriptional terminators or that are not co-localised with associated precursor-supplying genes or tailoring enzymes. The only example reported to our knowledge is the mycosporine-like amino acid BGC (*mys*) pathway in *Nostoc* sp. UHCC 0926, where biosynthetic genes reside in three genomically distant loci, necessitating a branched biosynthetic model to explain the production of two structurally distinct products [70]. By enabling precise reconstruction of fragmented loci, overlapping extension PCR reduces reliance on extensive native genomic context and reduces the need for multi-fragment DNA assembly reactions. Consequently, this strategy expands the range of BGC architectures that can be effectively captured and functionally characterised.

### Assembly of the desired expression construct

A wide range of cloning and expression vectors are available for BGC capture and since DiPaC relies predominantly on PCR-based amplification and sequence-independent assembly *in vitro*, the choice of vector backbone is generally flexible [26]. The vector of choice must be linearised prior to the assembly, which can be achieved either by PCR amplification or restriction digest. PCR-based linearisation is limited by vector size—an important consideration during stepwise assembly of increasingly large constructs. However, unique restriction sites introduced by primer sequence and then located at the 3′-end of the insert can be utilised for downstream vector linearisation. A subsequent DpnI digest is essential for PCR linearised vectors to remove the parental plasmid template and thereby improve transformation efficiency of the newly assembled construct. In contrast, restriction digest is size-independent, and technically straightforward but does not permit small edits to the plasmid sequence.

For the assembly of amplified BGC-DNA fragments with linear vector backbones, both HiFi Gibson Assembly or SLIC are well-established and optimal choices. Both approaches rely on enzymatic preparation of terminal-end ssDNA overhangs, enabling complementary homologous sequences on different strands to anneal. SLIC is often a cost-effective and rapid first option and relies on T4 DNA polymerase, which possess a 3′ to 5′ exonuclease activity in the absence of deoxynucleotide triphosphate substrates [71]. An important consideration is the exonuclease activity rate of T4 DNA polymerase being approximately 40 bp/min. Long reaction times will result in extended ssDNA overhangs and increase likelihood of secondary structures. Thus, reaction times of 150 seconds at room temperature are common but shorter reactions of 30 seconds at 37°C have also been found to be highly efficient [72,73]. The SLIC reaction is terminated by placing the mixture on ice and any remaining gaps, overhangs, or nicks are repaired *in vivo* post-transformation by *E. coli*. Functionally analogous to SLIC, HiFi DNA Assembly, an optimised implementation of the original Gibson Assembly method [30], uses a combination of enzymes, including a 5′ exonuclease to generate terminal ssDNA, DNA polymerase for extending 3′ overhangs, and DNA ligase to covalently seal gaps in the assembled construct in a single *in vitro* isothermal reaction, producing fully sealed constructs. The assembly reaction is then incubated at 50°C for 15–60 minutes prior to transformation into *E. coli*.

When considering SLIC versus HiFi DNA assembly, the formation of stable secondary structures at the terminal ends of the newly generated ssDNA has the largest impact on cloning efficiency. Thus, while SLIC is quicker and more cost efficient compared to HiFi Gibson Assembly, the lower reaction temperature and time (length of ssDNA) are important parameters that need to be considered. In particular, large, GC-rich, or structurally complex DNA fragments may assemble inefficiently and in such cases, HiFi Gibson Assembly may be preferable. However, in our experience either SLIC or HiFi DNA Assembly does not consistently outperform the other, and both techniques should be considered when capturing a BGC of interest.

For all cloning strategies, maintaining appropriate molar ratios is crucial: ideally, fragment-to-vector ratios should reflect their relative sizes, aiming for at least ∼0.02 pmol of each component. Following transformation, colony-screening PCR (e.g. using Taq polymerase) is recommended to identify preliminary positive clones. Furthermore, PCR control of the DNA assembly mixture can help distinguish assembly failure from poor transformation efficiency. Preliminary positive clones are recommended to be analysed by restriction digest to ensure expected construction of the entire plasmid followed by Sanger sequencing over assembled junctions to ensure correct assembly of the terminal ends of DNA or whole-plasmid sequencing. Proper positive and negative controls are essential to assess the competence of the cells used, and in difficult cases, freshly prepared competent cells may outperform frozen batches. Chemical transformation has been successfully used to propagate large plasmids but if problems persist, electroporation can improve efficiency, albeit requiring specialised equipment. Collectively, systematic optimisation of assembly conditions and transformation steps is key for reliable recovery of large or structurally complex genetic constructs (Figure 3).

## Conclusion and future directions

DiPaC provides a flexible platform for capturing, refactoring, and expressing BGCs, enabling rapid access to BGCs across a wide range and from diverse NP classes. Its reliance on long-range PCR and *in vitro* assembly allows efficient cloning from minimal DNA material while providing freedom in vector choice and pathway design. This versatility has facilitated the discovery of numerous new metabolites, clarified ambiguous BGC architectures, and supported studies ranging from biosynthetic elucidation to compound structural diversification and optimisation of production titers. DiPaC thus helps bridge the gap between bioinformatically predicted BGCs and experimental validation.

Looking ahead, the growing accessibility and decreasing cost of synthetic DNA are expected to further transform the landscape of BGC cloning and engineering, allowing greater access to unculturable organisms. As *de novo* DNA synthesis becomes increasingly feasible for large and complex BGCs, DiPaC-like hybrid approaches combining direct amplification of native DNA with synthetic reconstruction of problematic regions are poised to further expand the boundaries of what can be functionally explored. Together, these developments position DiPaC as a cornerstone strategy in modern NP research, with lasting relevance for the systematic exploration, engineering, and exploitation of microbial biosynthetic diversity as both sequencing and synthesis technologies mature.

## Summary

- The gap between bioinformatically predicted cryptic BGCs and functional characterisation represents a bottleneck in NP discovery, necessitating efficient cloning and heterologous expression strategies.
- A key advantage of DiPaC is the ability to modify BGC architecture during the cloning process, allowing removal of problematic regulatory elements, promoter exchange, and reorganisation of genes without requiring post-capture engineering steps or additional antibiotic selection markers.
- The DiPaC strategy has successfully been applied to activate BGCs of small to medium size sourced from different bacterial phyla and NP classes
- Practical implementation requires systematic planning of refactoring strategies, careful optimisation of PCR conditions for challenging templates, and selection of appropriate assembly approaches.
- As synthetic DNA becomes increasingly accessible, DiPaC workflows will likely evolve to seamlessly combine native sequences with synthetic variants, further facilitating discovery efforts.

## Open Access

Open access for this article was enabled by the participation of Saarland University in an all-inclusive Read & Publish agreement with Portland Press and the Biochemical Society under an agreement with the TIB German Alliance.

## Abbreviations

BGC: Biosynthetic gene cluster
BLAST: Basic Local Alignment Search Tool
CDS: Coding DNA sequence
DiPaC: Direct Pathway Cloning
gDNA: Genomic DNA
NCBI: National Center for Biotechnology Information
NEB: New England Biolabs
NP: Natural product
NRPS: Non-ribosomal peptide synthetase
ORF: Open reading frame
PKS: Polyketide synthase
PoTeM: Polycyclic tetramate macrolactam
RiPP: Ribosomally synthesised and post-translationally modified peptide
SLIC: Sequence- and ligation-independent cloning
ssDNA: Single-stranded DNA
Tm: DNA melting temperature
